# On the Instrumental Discrepancies in Lyman-Alpha Observations of Solar Flares

**DOI:** 10.1007/s11207-024-02407-7

**Published:** 2024-11-27

**Authors:** Harry J. Greatorex, Ryan O. Milligan, Ingolf E. Dammasch

**Affiliations:** 1 Astrophysics Research Centre, School of Mathematics and Physics, Queen’s University Belfast, Belfast, BT7 1NN UK; 2 Solar Influences Data Analysis Center, Royal Observatory of Belgium, Circular Avenue 3, 1180 Uccle, Brussels, Belgium

## Abstract

Despite the energetic significance of Lyman-alpha (Ly$\alpha $; 1216 Å) emission from solar flares, regular observations of flare related Ly$\alpha $ have been relatively scarce until recently. Advances in instrumental capabilities and a shift in focus over previous solar cycles mean it is now routinely possible to take regular co-observations of Ly$\alpha $ emission in solar flares. Thus, it is valuable to examine how the instruments selected for flare observations may influence the conclusions drawn from the analysis of their unique measurements. Here, we examine three M-class flares each observed in Ly$\alpha $ by GOES-14/EUVS-E, GOES-15/EUVS-E, or GOES-16/EXIS-EUVS-B, and at least one other instrument from PROBA2/LYRA, MAVEN/EUVM, ASO-S/LST-SDI, and SDO/EVE-MEGS-P. For each flare, the relative and excess flux, contrast, total energy, and timings of the Ly$\alpha $ emission were compared between instruments. It was found that while the discrepancies in measurements of the relative flux between instruments may be considered minimal, the calculated contrasts, excess fluxes, and energetics may differ significantly – in some cases up to a factor of five. This may have a notable impact on multi-instrument investigations of the variable Ly$\alpha $ emission in solar flares and estimates of the contribution of Ly$\alpha $ to the radiated energy budget of the chromosphere. The findings presented in this study will act as a guide for the interpretation of observations of flare-related Ly$\alpha $ from upcoming instruments during future solar cycles and inform conclusions drawn from multi-instrument studies.

## Introduction

The Lyman-alpha line of neutral hydrogen (Ly$\alpha $; 1216 Å) is the most intense emission line in the quiescent solar spectrum (Curdt et al. 2001). In spite of this, observations of flare-related Ly$\alpha $ emission have historically been relatively scarce. Among the earliest measurements of flare-related Ly$\alpha $ emission were the spectroscopic observations of Skumanich et al. (1978) using the *Laboratoire de Physique Stellaire et Planetaire* (LPSP: Bonnet et al. 1978) onboard *Eighth Orbiting Solar Observatory* (OSO-8), and of Canfield and van Hoosier (1980) using the NRL Spectrograph (Bartoe et al. 1977) within the *Apollo Telescope Mount* onboard *Skylab*. However, due to the individual instrumental capabilities required to observe such an energetic emission line on flare timescales, it has only been possible in the last decade to conduct statistical studies using calibrated photometric measurements of flare-related Ly$\alpha $.

Milligan et al. (2020) conducted a large-scale statistical study of Ly$\alpha $ flares observed by the *Geostationary Operational Environmental Satellites* (GOES) during Solar Cycle 24. From their analysis of $>500$ M- and X-class flares, it was found that both the energy and contrast in flare-related Ly$\alpha $ emission tend to scale with Soft X-Ray (SXR) magnitude. Furthermore, it was found that 95% of the flares sampled had an associated Ly$\alpha $ contrast of $<10\%$. However, unique cases could reach contrasts of up to $\sim 30\%$. Comparatively, observations of flare-related Ly$\alpha $ from the *Project for On-Board Autonomy* (PROBA2: Santandrea et al. 2013) have consistently found contrasts of $\mathrm{<1\%}$ (Kretzschmar, Dominique, and Dammasch 2013; Raulin et al. 2013). One explanation for these notably low contrasts may be significant detector degradation and the presence of contaminants in the PROBA2 signal. Currently, the extent to which these discrepancies may exist in co-observations between individual instruments is not well documented.

Ly$\alpha $ emission is optically thick and therefore observations in Ly$\alpha $ are subject to effects of Centre-to-Limb Variation (CLV), whereby the position of a flare on the solar disk (and the subsequent column depth along the observing line of sight) can impact the measured Ly$\alpha $ irradiance (for further discussion, see Woods et al. 1995; Woods, Kopp, and Chamberlin 2006; Milligan 2021). The extent to which CLV may impact Ly$\alpha $ observations was examined by Milligan et al. (2020) using combined stereoscopic observations of an X1.1 flare from GOES-15 and the *Mars Atmosphere and Volatile Evolution* (MAVEN: Eparvier et al. 2015) in orbit around Mars. For MAVEN, the flare was located close to disk-centre, whereas for GOES the flare appeared at the solar limb. Subsequently, a $\sim 45\%$ increase in flare-related Ly$\alpha $ excess was observed at Mars relative to the Earth, thus demonstrating the magnitude of CLV on observations of optically-thick emission.

Temporally, flare-related Ly$\alpha $ emission tends to peak in conjunction with nonthermal Hard X-Ray (HXR) emission and therefore the derivative of the SXRs in alignment with the Neupert Effect (Neupert 1968); examples of the nonthermal origin of Ly$\alpha $ emission are demonstrated in Nusinov et al. (2006), da Costa et al. (2009), Milligan et al. (2017), Dominique et al. (2018), Li et al. (2022), Tian et al. (2023), and Greatorex, Milligan, and Chamberlin (2023). In some instances, secondary Ly$\alpha $ emission peaks occur during the decay-phase, which is speculated to originate from cooling loop plasma rather than the flare footpoint regions (Kretzschmar, Dominique, and Dammasch 2013), or from filament eruptions (Wauters et al. 2022). However, systematic effects have also been shown to account for apparent violations of expected Neupertian behaviour. Milligan and Chamberlin (2016) found flare-related Ly$\alpha $ emission observed by the *Extreme-ultraviolet Variability Experiment* (EVE: Woods et al. 2012) onboard SDO to peak within the gradual phase co-temporally with the associated Soft X-ray (SXR) emission. This was later attributed to issues with the Kalman filters used during data processing (D. Woodraska – private communication).

Recent instrumental advancements mean that it is now possible to image solar flares in Ly$\alpha $ on sufficient timescales to observe the dynamic spatial development of flare footpoints, loops, and ribbons. Li et al. (2022) studied a C1.4 flare using observations from the Ly$\alpha $ channel of *High Resolution Imager* within the *Extreme Ultraviolet Imager* onboard *Solar Orbiter* (SO/EUI-HRI_Ly*α*_: Müller et al. 2020; Rochus et al. 2020,1). In their study, the authors found a co-spatial relationship between Ly$\alpha $ emission and nonthermal HXR sources, similarity in spatiotemporal behaviour between Ly$\alpha $ and He ii (304 Å) emission observed by the *Extreme Ultraviolet Imager* (EUVI: Howard et al. 2008) onboard the *Solar Terrestrial Relations Observatory Ahead* (STEREO-A: Kaiser et al. 2008) and the *Atmospheric Imaging Assembly* onboard the *Solar Dynamics Observatory* (SDO/AIA: Pesnell, Thompson, and Chamberlin 2012; Boerner et al. 2012), and Ly$\alpha $ brightenings in both the rise and decay phase of the flare. Furthermore, the *Solar Disk Imager* within the *Lyman-alpha Solar Telescope* onboard the recently launched *Advanced Space-based Solar Observatory* (ASO-S/SDI-LST: Li et al. 2019; Feng et al. 2019; Gan et al. 2023; Chen et al. 2024) has been used to examine the presence of long-period QPPs in an X6.4 solar flare, co-temporal with the associated HXR emission (Li et al. 2024). Similar behaviour was presented by Milligan et al. (2017) in a multi-instrument study using observations from GOES, SDO, and the *Reuven Ramaty High Energy Solar Spectroscopic Imager* (RHESSI: Lin et al. 2002).

Multi-instrument observations are crucial for examining fundamental open questions surrounding the origin of flare-related emission, constraining the radiated energy budget of the chromosphere, and the temporal evolution of flare-related emission during different phases of solar flares. Using joint observations from SDO, GOES, PROBA2, and RHESSI, Wauters et al. (2022) examined an M6.7 flare with an additional peak in Ly$\alpha $ emission during the decay phase of the flare that had no temporal correlation to the HXR or SXR emission. Using images in 1600Å from SDO/AIA, the source of the emission was later attributed to a failed filament eruption in proximity to the flare origin. Moreover, using combined observations from RHESSI and GOES, both Milligan et al. (2014) and Greatorex, Milligan, and Chamberlin (2023) were able to examine the contribution of Ly$\alpha $ to the radiated energy budget of the chromosphere during solar flares. These studies found for both an X- and M-class flare, Ly$\alpha $ may account for up to $\sim 8\%$ of the energy deposited into the chromosphere by nonthermal electrons.

When conducting precise analysis of solar flares, it is currently unclear to what extent the instruments chosen for observations may impact the conclusions drawn from their observations. This fact becomes more pertinent with the consideration of newly-launched and upcoming missions such as ASO-S, GOES-R, and Solar-C (Watanabe 2014). With the wealth of available data from current missions, it is possible to thoroughly examine Ly$\alpha $ emission during solar flares. This paper aims to assess the level of agreement in Ly$\alpha $ observations of solar flares between different missions, focussing on observable metrics that may be used to infer the underlying mechanisms driving the emission, such as the relative and excess fluxes, the flare contrasts, energetics, and timings. This analysis should act as a guide for future studies using next generation instruments.

The instruments examined within this study are summarised in Section 2. A discussion of the unique instrumental observing capabilities including the spectral response functions and bandpasses is presented in Section 3. The flare sample used for the individual case studies, as well as the calibration, standardisation, and analysis techniques are presented in Section 4. Section 5 details the results of each case study. Finally, a discussion of the impact of this study and future Ly$\alpha $ observation capabilities is presented in Section 6.

## Instrumentation

This study focuses on the examination of observations of flare-related Ly$\alpha $ emission from multiple missions that have served the solar community over Solar Cycles 24 and 25. The following section presents a brief summary of each instrument included in this study and details the individual calibration processes carried out for each instrument where relevant. Table 1 contains a technical summary of each instrument.

**Table 1:** Summary of the observing instruments from each mission used in this work. *Cadences are taken as the cadence of the L2 calibrated data available from the dedicated mission repositories for each observation. **Average between two peaks in GOES-15/EUVS-E response function.

Mission	Instrument	Orbit	Observation Type	Cadence (s)*	Bandpass FWHM (Å)	Response Peak *λ* (Å)
GOES-14	EUVS–E	Geostationary	Photometry	10.24	131.1	1214
GOES-15	EUVS–E	Geostationary	Photometry	10.24	108.7	1227**
GOES-16	EXIS–EUVS–B	Geostationary	Photometry	60.0	–	–
PROBA2	LYRA	Sun-synchronous	Photometry	0.05	104.5	1200
MAVEN	EUVM	Areocentric	Photometry	1.0	64.4	1200
SDO	EVE-MEGS-P	Geosynchronous	Photometry	60.0	91.6	1205
ASO-S	LST-SDI	Sun-synchronous	Imaging	60.0	92.3	1216

### GOES-14/EUVS–E and GOES-15/EUVS–E

GOES-14 and GOES-15 both featured an *X-Ray Sensor* (XRS: Hanser and Sellers 1996; Chamberlin et al. 2009) and *Extreme Ultraviolet Sensor* (EUVS: Viereck et al. 2007; Evans et al. 2010), observing disk-integrated SXR and Extreme Ultraviolet (EUV) emission at a near 100% duty cycle. EUVS consisted of five channels (A–E) covering the 50 – 170, 240 – 340, 200 – 620, 200 – 800, and 1180 – 1250 Å wavelength ranges. The E-channel (EUVS–E) was a dedicated broadband channel centred around the Ly$\alpha $ line at 1216 Å, observing the full solar disk at 10.24 s cadence. These GOES satellites operated in a geostationary orbit at an altitude of approximately 36 000 km. Thus, observations from GOES are subject to geocoronal absorption that are more pronounced around the equinoxes. In this instance, EUV emission is absorbed by hydrogen in the geocorona, leading to a reduction in the observed emission. As a result, it is important to consider Ly$\alpha $ observations where the effect of the geocorona is minimal and/or can be accounted for. The GOES-14/EUVS-E and GOES-15/EUVS-E observations suffer from degradation over time, which is compensated for by using daily averages from the *Solar Stellar Irradiance Comparison Experiment* onboard the *Solar Radiation and Climate Experiment* (SORCE/SOLSTICE: McClintock, Rottman, and Woods 2005) to scale the daily averages from GOES. Thus, while the degradation and absolute values are scaled to SORCE/SOLSTICE, the variability is determined by the GOES instrument. For this study, the degradation corrected 1 nm band (121 – 122 nm) data were used.

### GOES-16/EXIS–EUVS–B

GOES-16 operates with an incorporated set of SXR and EUV sensors as part of a combined *Extreme Ultraviolet and X-ray Irradiance Sensors* (EXIS) suite. The EUVS for EXIS is comprised of two channels (A and B) measuring the spectral irradiance of lines within the 250 – 310 and 1170 – 1410 Å wavelength ranges, respectively, and a third channel (Channel-C) taking relative measurements of the Mg ii core/wing ratio between 2750 – 2850 Å. The EUVS–B channel comprises a photodiode array with diode clusters allocated to cover significant solar emission lines such as C iii (1175 Å), H i Ly$\alpha $ (1216 Å), C ii (1335 Å), and S iv/O iv (1405 Å), each with an approximate width of 6 Å. The final irradiance data from EUVS-B is a summation of the fluxes over the photodiodes for each separate cluster. At the time of writing, these full-disk L2 irradiances from EXIS/EUVS–B are available at 60 s cadence, future products following additional processing are expected to have cadences of 1 s. Similar to GOES-14/15, GOES-16 operates in a geostationary orbit with an altitude of approximately 36 000 km (see Eparvier et al. 2009 for a detailed discussion of EUVS for GOES-16). For this study, Version 1.0.5 of the science-quality solar line data from GOES-16/EXIS were used; at the time of writing this is the most up-to-date publicly available version of the GOES-16 data.

Several caveats need to be considered for GOES-16/EXIS-EUVS-B data. First, the presence of multi-hour post-eclipse thermal dips in the spectral lines due to incompletely corrected temperature impacts may affect the EUVS observations, although it is simple to exclude these data as required. The magnitude of this in the Ly$\alpha $ line is difficult to quantify due to the additional effect of the geocorona. Furthermore, an annual cycle oscillation artifact with a magnitude of 1.3% of the measured flux impacts the irradiances of the Ly$\alpha $ channel of EUVS. It is unclear what impact these may have on the observations presented in this study.

### PROBA2/LYRA

The *Large Yield Radiometer* (LYRA: Hochedez et al. 2006; Dominique et al. 2013) onboard PROBA2 is a tri-unit broadband radiometer with four distinct channels observing in the SXR to mid-ultraviolet regime. The Ly$\alpha $ filters from Units 1 and 2 (MSM Diamond Detector) and Unit 3 (AXUV Si Detector) of LYRA cover the 1200 – 1230 Å wavelength range, taking full-disk irradiance measurements at 0.05 s cadence. The nominal unit of LYRA has suffered significant degradation and no longer observes Ly$\alpha $ emission, due to changes in the Ly$\alpha $ channel bandpass. The degradation of the LYRA instrument has been attributed to the deposition of carbon and silicon on the detectors, creating a contaminant layer that is more opaque to longer wavelengths relative to short resulting in different signal losses depending on wavelength (BenMoussa et al. 2015; Wauters et al. 2022). The data collected during special observing campaigns using the backup unit are still sufficient to observe flare related Ly$\alpha $ emission (M. Dominique 2023 – private communication). PROBA2 has a Sun-synchronous orbit at 720 km altitude placing it in the Low-Earth Orbit.

### MAVEN/EUVM

The *Extreme Ultraviolet Monitor* (EUVM) onboard the MAVEN satellite is a Sun-facing instrument consisting of three channels taking disk-integrated broadband irradiances in the SXR and EUV regimes. The Ly$\alpha $ channel (Channel C) of EUVM covers the 1170 – 1250 Å wavelength range, measuring irradiance at 1 s cadence. Nominal EUVM observations are taken in the operations case with the Sun within science FOV and aperture mechanism “open”, which produces valid solar irradiance measurements. MAVEN operates in a highly elliptical orbit around Mars, with an apoapsis of ∼6000 km and periapsis of ∼150 km.

### SDO/EVE-MEGS-P

SDO/EVE features a set of *Multiple EUV Grating Spectrographs* (MEGS) comprised of three main components. The grazing-incidence spectrograph (MEGS-A) and normal-incidence spectrograph (MEGS-B) sample the 50 – 370 and 350 – 1050 Å wavelength range at 1 Å resolution, respectively.^2^ The broadband photodiode (MEGS-P) used with the first grating of MEGS-B is centred on the Ly$\alpha $ line. SDO operates in a 28^∘^ inclined geosynchronous orbit at an altitude of 36 000 km. At the time of writing, the degradation of MEGS-B means that MEGS-P operates on a flare trigger system, with a 60 s cadence (previously MEGS-P has operated with 10 s cadence).

### ASO-S/LST-SDI

The ASO-S mission is the first space-based Chinese mission dedicated to solar observation. The LST is one of the instruments onboard ASO-S. The SDI within LST images the full solar disk at 1216 Å with a spatial resolution of $9.5''$ and a routine exposure time of 13.5 s.^3^ ASO-S operates in a Sun-synchronous orbit with an altitude of 720 km and an inclination of 98^∘^ relative to the Earth’s equator. The nominal cadence for routine SDI observations is between 4 – 40 s; SDI images are also available in 60 s intervals due to the “*image download interval*” set in response to telemetry limitations.

## Spectral Response Functions and Bandpasses

**Figure 1:**
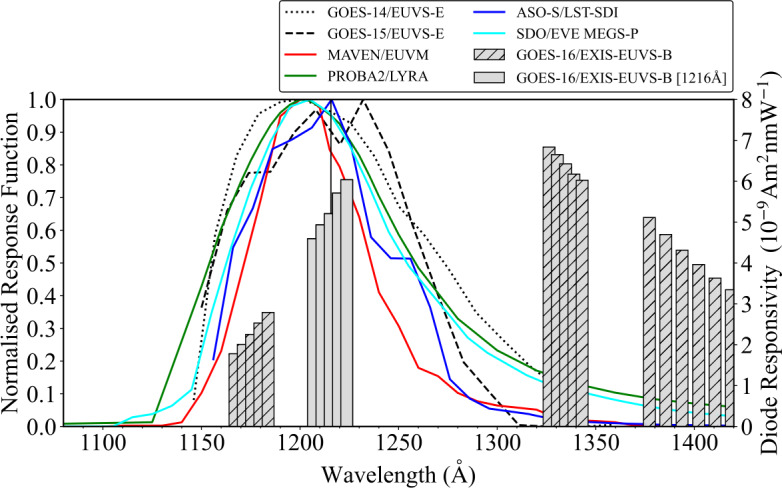
Normalised spectral responses for the instruments examined in this work. The Ly$\alpha $ line core at 1216 Å is denoted by the vertical solid line. The GOES-16 diodes are denoted by grey bars, where the width of the bar is approximately 6 Å. Unhatched grey bars denote the cluster about the Ly$\alpha $ line.

The spectral response of a given instrument quantifies the effectiveness of an instrument in transforming incident flux into a measurable output for a given emission spectrum. Figure 1 shows the normalised spectral response for each broadband instrument described in Section 2. Also presented are the diode responsivities of GOES-16/EXIS–EUVS–B, where each grey bar denotes a unique photodiode. Generally, the normalised spectral responses for the broadband Ly$\alpha $ instruments resemble a Gaussian profile with the central peak residing within the 1200 – 1220 Å wavelength range. It appears that several of the spectral responses are centred slightly blueward of the Ly$\alpha $ core at 1216 Å (denoted by the vertical solid black line in Figure 1). The GOES-15/EUVS–E spectral response appears to be double peaked, with the larger peak appearing around 1245 Å and the average of the two peaks being ∼1227 Å. The responsivities for GOES-16/EXIS–EUVS–B show the distinct clusters centred about their unique corresponding emission lines, with cluster B positioned about the Ly$\alpha $ core and the responsivity apparently increasing monotonically with wavelength. It should be noted that the responses are normalised for comparison and do not necessarily give an indication of the true instrumental responsivity as they are dimensionless. The true responsivity may change due to degradation over the instrument lifetime. The wavelength at the peak of the spectral response for each instrument is summarised in Table 1.

**Figure 2:**
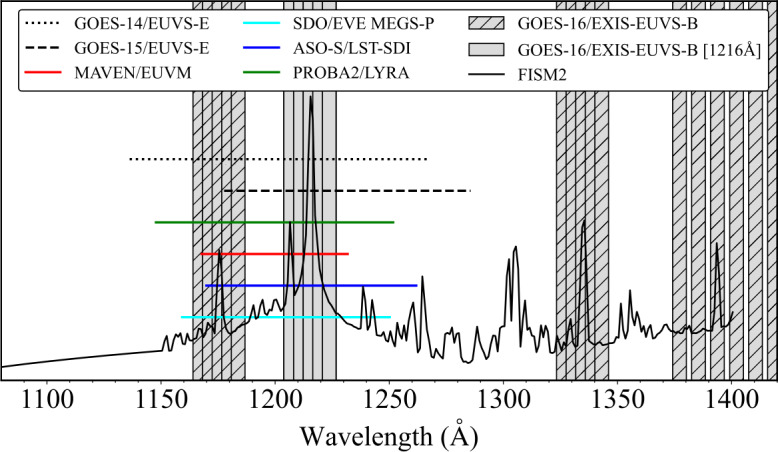
FWHM of the spectral responses presented in Figure 1. The horizontal lines denote the widths of the FWHM bandpasses. The GOES-16 diodes are denoted by grey bars, where the width of each bar is approximately 6 Å. Unhatched grey bars denote the cluster about the Ly$\alpha $ line. A QS EUV spectrum from FISM2 is overplot in the solid, thin black line.

The FWHM of the spectral response function for each instrument is presented as a horizontal line in Figure 2. Also included is a model of a daily averaged quiet-Sun (QS) EUV spectrum from the *Flare Irradiance Spectral Model v2* (FISM2: Chamberlin et al. 2020a), where the prominent central peak corresponds to the Ly$\alpha $ line. The values of the FWHM bandpasses are summarised in Table 1. From Figure 2, GOES-14/EUVS–E has the broadest bandpass and MAVEN/EUVM has the narrowest. In terms of FWHM values, the values between instruments show reasonable similarity. However, the central point of the bandpass appears to demonstrate a non-negligible difference between each instrument.

**Figure 3:**
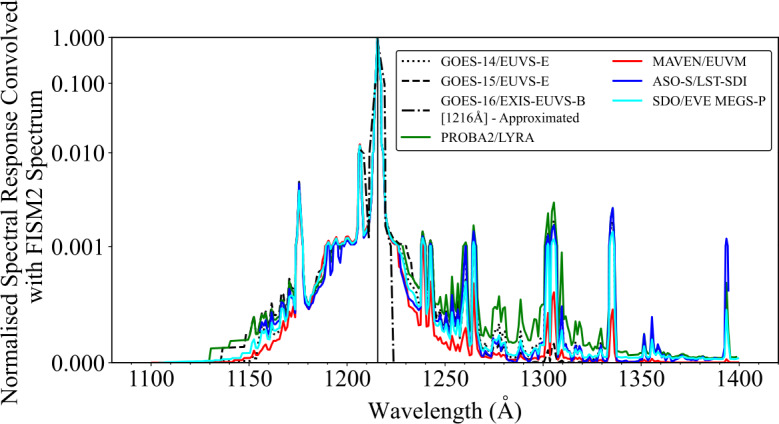
Convolution of a model QS EUV spectrum from FISM2 and the spectral responses for each instrument on a logarithmic scale. The black dotted–dashed line denotes a rough approximation of the GOES-16/EXIS-EUVS-B response about the Ly$\alpha $ cluster derived from the diode responsivities.

To explore the spectral purity of the observations, the spectral response for each instrument was convolved with a model QS spectra from FISM2 in the 1080 – 1420 Å wavelength range (Figure 3). From this convolution, it is apparent that for all the instruments examined the measured irradiance across the full bandpass is dominated by Ly$\alpha $. From Figure 3 it is apparent that contributions to the irradiance from nearby species such as Si iii (1206 Å) and O v (1218 Å) are found to be 100 – 1000 times less than that of Ly$\alpha $, thus suggesting it is reasonable to assume that the observed irradiance from each instrument is dominated by Ly$\alpha $ (Woods et al. 2012 state the filter purity of MEGS-P to be 99%).

## Flare Sample and Analysis

In order to make instrumental comparison between flare observations, three case studies were conducted each using a unique combination of instruments. The flare sample used for these studies was selected on the fulfillment of the following criteria:

- Each flare was M-class or above to facilitate sufficient observable Ly$\alpha $ flux increases attributed to the flare.
- Each flare was co-observed by the active GOES satellite at the time of the observation and at least one other instrument from Section 2.
- Each flare occurred on-disk from the respective instrument FOV, reducing the impact of Centre-to-Limb Variation.
- The full flare period was observed by each instrument examined in that case study, with a sufficient preflare period to calculate a background flux value.
- Instruments must be operating in standard observation modes to prevent additional impact of increased cadences, additional attenuation from filters, spacecraft maneuvers, and variable pointing (imagers, etc.).

Table 2 presents a summary of three flares that met the above criteria. The flare timings and classes were taken from the 1 – 8 Å SXR observations from the XRS instrument on the relevant GOES satellite.

**Table 2:** Observational summary of the flare sample.

Solar Object Locator	GOES Start (UT)	GOES Peak (UT)	GOES End (UT)	GOES Class	Heliographic Position	Observing Instrument
SOL2010–02–08	13:32	13:47	13:50	M2.0	N28W08	GOES-14/EUVS–E
						PROBA2/LYRA
SOL2016–04–18	00:14	00:29	00:39	M6.7	N11W60	GOES-15/EUVS–E
						MAVEN/EUVM
SOL2023–05–09	03:42	03:54	04:05	M6.5	N13W26	GOES-16/EXIS–EUVS–B
						ASO-S/LST-SDI
						SDO/EVE-MEGS-P

### Standardisation and Calibration

In order to make a reasonable comparison between instruments, Ly$\alpha $ measurements were converted to disk-integrated irradiance at 1 AU, in units of $\mathrm{Wm^{-2}}$. The following sections detail the calibration and standardisation procedures conducted for the relevant instruments.

#### Scaling Observations to 1 AU

As MAVEN is a Mars-orbiting mission, a scaling factor is required to convert the Ly$\alpha $ observations from Mars to the Earth distance. This scaling factor is calculated as $\mathrm{(\frac{R_{MS}}{R_{ES}})^{2}}$, where $\mathrm{R_{MS}}$ and $\mathrm{R_{ES}}$ are the Mars–Sun and Earth–Sun separation distances at the time of the flare, respectively. A scaling factor of 2.52 was found for MAVEN.

Additionally, a light travel-time correction factor was calculated as:

1 $$ \Delta t \ = \ \frac{R_{\mathrm{ScS}} - R_{\mathrm{ES}}}{c} \ \mathrm{{seconds}}, \mathrm{ }$$

where c is the speed of light in m s^−1^ and the separation distance is given in m. This Earth–Mars travel time could then be subtracted from the observation time to align with observation time taken at 1 AU. The calculated value of $\Delta \mathrm{t}$ for MAVEN at the time of the flare was found to be 289 s. Similar scaling was performed for Earth-orbiting instruments to ensure all observations were scaled to 1 AU.

#### Radiometric Calibration of Images

Image data require radiometric calibration in order to covert the pixel data in $\mathrm{DNs^{-1}}$ to irradiance in $\mathrm{Wm^{-2}}$. For ASO-S/LST-SDI, this calibration was carried out using the IDL program lst_radcalib.pro within the LST analysis package available from the *Science Operation and Data Centre* (SODC). For this calibration, the image data were despiked using “*L.A. Cosmic*” cosmic ray identification algorithm (van Dokkum, Bloom, and Tewes 2012), incorporated into the LST software package, and a Radiometric Calibration Factor (RCF) of $1.98 \cdot 10^{-8}\ \mathrm{erg~DN^{-1}~cm^{-2}}$ was applied. The RCF is calculated using the ratio of EUVS Ly$\alpha $ daily averages from GOES to LST data and is time-dependent due to degradation of instrument over the mission lifetime. Following this, image data from ASO-S/LST-SDI was converted from spatially-resolved data to 1-dimensional lightcurves via summation of the pixels present in the full-disk image. It was assumed that the flux within the image exposure time may be extrapolated across the total imaging cadence. This may have an impact on the understanding of the temporal behaviour of the flux, although will not impact the magnitude of Ly$\alpha $ emission.

#### Centre-to-Limb Variation Correction

**Figure 4:**
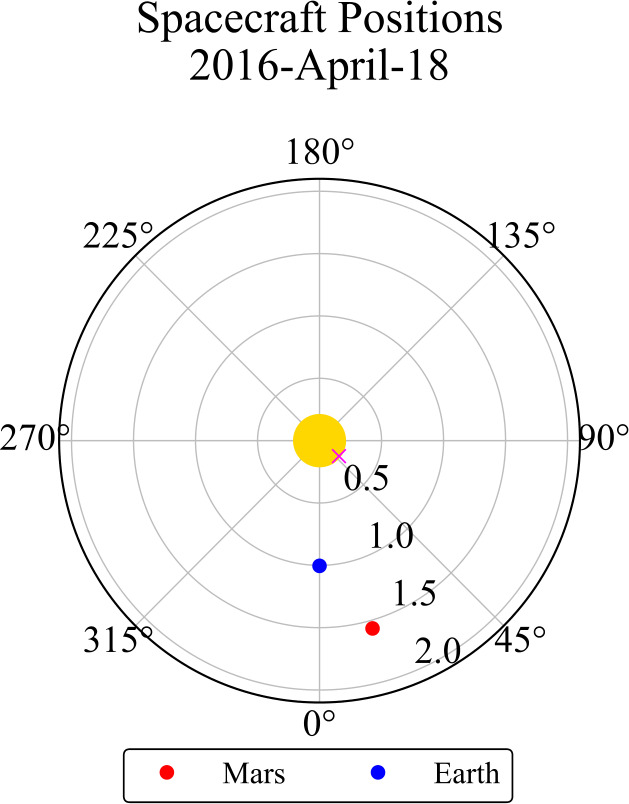
Approximate position relative to the Sun–Earth line for Mars in Heliographic Stonyhurst coordinates on the date of the flare observation. The magenta cross marks the approximate position of SOL2016–04–18. Labelled concentric circles denote distance from the Sun (central yellow circle) in AU.

Given the position of SOL2016–04–18 on the solar disk, and the differing vantage points of GOES-15 and MAVEN, a CLV correction factor was required to account for the impact of the relative flare position on the observed Ly$\alpha $ emission. First, a translation of the longitudinal flare position was performed from the position on-disk as seen from the Earth to the approximate position as seen from Mars. This was carried out under the assumption of an unchanged flare latitude via the following:

2 $$ \phi _{\mathrm{Mars}} \ = \ \phi _{\mathrm{Earth}} - \Delta \theta _{\mathrm{Earth }-\mathrm{ Mars}} ,$$

where $\phi _{\mathrm{Mars}}$ and $\phi _{\mathrm{Earth}}$ denote the approximate longitudes as observed from Mars and the Earth, respectively, and $\Delta \theta _{\mathrm{Earth }-\mathrm{ Mars}}$ denotes the angular separation between the Earth and Mars at the time of the flare. The position of Mars relative to the Earth at the time of SOL2016–04–18 is presented in Figure 4. The value of $\Delta \theta _{\mathrm{Earth }-\mathrm{ Mars}}$ was found to be 15.8^∘^. From Equation 2, the angle $\phi _{\mathrm{Mars}}$ was calculated as $\sim 44^{\circ}$, thus giving approximate heliographic coordinates of SOL2016–04–18 relative to Mars of N11W44.

Following this, a CLV correction factor (C) was calculated for both vantage points using a cosine-squared method, which is justified by the fall-off of irradiance toward the solar limb as demonstrated by Milligan et al. (2020). The CLV correction factor is calculated as

3 $$ C = \cos ^{2}(\omega ) \left (1 - \frac{|\phi |}{360}\right ) , $$

where $\omega $ is the heliographic latitude in degrees and $\phi $ is the heliographic longitude in degrees. This yielded CLV correction factors for the MAVEN and GOES-15 observations of 0.8030 and 0.8512, respectively. The measured Ly$\alpha $ fluxes from each instrument were then multiplied by their respective correction factors.

#### Recalibration of PROBA2/LYRA Data

The PROBA2/LYRA observations for SOL2010–02–08 were studied in detail by Kretzschmar, Dominique, and Dammasch (2013). In their study, the authors note that the standard degradation correction may underestimate the flare irradiance increase. Instead, they opted to manually calibrate the L1 data, correcting for degradation, dark currents, and spacecraft motion. The Ly$\alpha $ observations from PROBA2/LYRA were recalibrated using the methods from Kretzschmar, Dominique, and Dammasch (2013) to reproduce their observations for this study. Dark currents were estimated as a function of temperature and removed from the irradiance. A multiplicative degradation correction was applied by dividing the flux by the estimated (approximately linear) degradation for 8 February 2010. Finally, an orbital correction was applied by constructing an orbital pattern for 8 February 2010 and dividing the flux by this. The observations were also degraded to 3 s cadence by summation, thus providing an improved signal-to-noise ratio (SNR).

### Flare Analysis

For each flare, the preflare background was taken as the mean flux over a 10-minute period before the XRS start time. The flare contrast was calculated from the peak flux divided by the background, while the excess flux was taken as the background subtracted irradiance. The energy radiated in Ly$\alpha $ was found by converting the background subtracted irradiance from flux measured at the Earth ($\mathrm{I_{Earth}}$) to power radiated at the Sun ($\mathrm{P_{Sun}}$) by the following:

4 $$ P_{\mathrm{Sun}} \ = \ 2\pi R^{2}\cdot 10^{7}~I_{\mathrm{Earth}} \ \mathrm{erg~s^{-1} } ,$$

where $R$ is the Sun–Earth distance and all observations were scaled to 1 AU. The value $10^{7}$ is a conversion factor from J to erg. The total energy for each flare was then calculated by integrating the power between the GOES X-ray (XRS 1 – 8 Å) start and end times. The uncertainty in the observed fluxes was taken as the standard deviation of the flux in the preflare background period. Finally, the time of the measured Ly$\alpha $ peak for each observation relative to the GOES X-ray peak was calculated as $\mathrm{t^{SXR}_{Peak} }-\mathrm{ t}^{\mathrm{Ly}\alpha}_{\mathrm{Peak}}$ ($\Delta \mathrm{t_{Peak}}$) for each instrument. The uncertainty in peak time was taken as $\pm \mathrm{\frac{cadence}{2}}$.

## Results

Each case presented below details flare observations in Ly$\alpha $ from two or more instruments, examining the relative flux in Ly$\alpha $, the flare-related contrast, the excess flux, energetics, and timing. A quantitative summary of these observations is presented in Table 3.

**Table 3:** Summary of the analysis results for each observation. Relative flux peak values were taken from calibrated irradiances that were scaled to 1 AU and given to the nearest 2 significant figures.

Flare Identifier	Observing Instrument	Peak Flux_Rel_ (10^−3^ Wm^−2^)	Peak Contrast (%)	Peak Flux_Exc_ (10^−4^ Wm^−2^)	Total Energy (10^29^ erg)	$\mathrm{t}^{\mathrm{SXR}}_{\mathrm{Peak}}-\mathrm{t}^{\mathrm{L}y\alpha}_{\mathrm{Peak}}$ (s)
SOL2010–02–08	GOES-14/EUVS–E	7.1 ± 0.009	3.5 ± 0.1	2.4 ± 0.09	1.3 ± 0.001	129.6 ± 5.12
	PROBA2/LYRA	6.7 ± 0.01	0.7 ± 0.2	0.5 ± 0.1	0.4 ± 0.00007	129.0 ± 1.5
SOL2016–04–18	GOES-15/EUVS–E	7.3 ± 0.008	4.4 ± 0.1	3.2 ± 0.08	3.0 ± 0.001	249.2 ± 5.12
	MAVEN/EUVM	7.6 ± 0.007	7.4 ± 0.1	5.0 ± 0.007	5.5 ± 0.0001	248.1 ± 0.5
SOL2023–05–09	GOES-16/EXIS–EUVS–B	8.9 ± 0.01	0.7 ± 0.1	0.7 ± 0.01	0.3 ± 0.009	120.0 ± 30.0
	SDO/EVE-MEGS-P	9.5 ± 0.04	3.5 ± 0.5	3.2 ± 0.04	4.3 ± 0.04	120.0 ± 30.0
	ASO-S/LST-SDI	9.2 ± 0.01	1.6 ± 0.1	1.4 ± 0.01	2.0 ± 0.009	104.0 ± 30.0

### SOL2010–02–08

**Figure 5:**
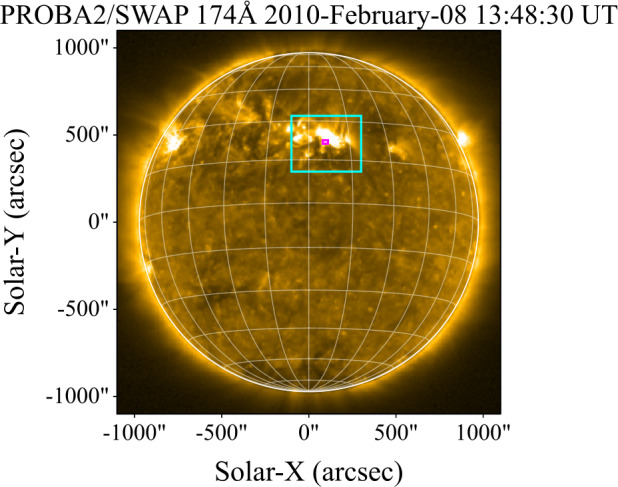
PROBA2/SWAP (174 Å) image of SOL2010–02–08 at a time close to the flare peak. The cyan bounding box denotes the active region attributed to the solar flare. The small magenta box denotes the approximate position of the flare source.

**Figure 6:**
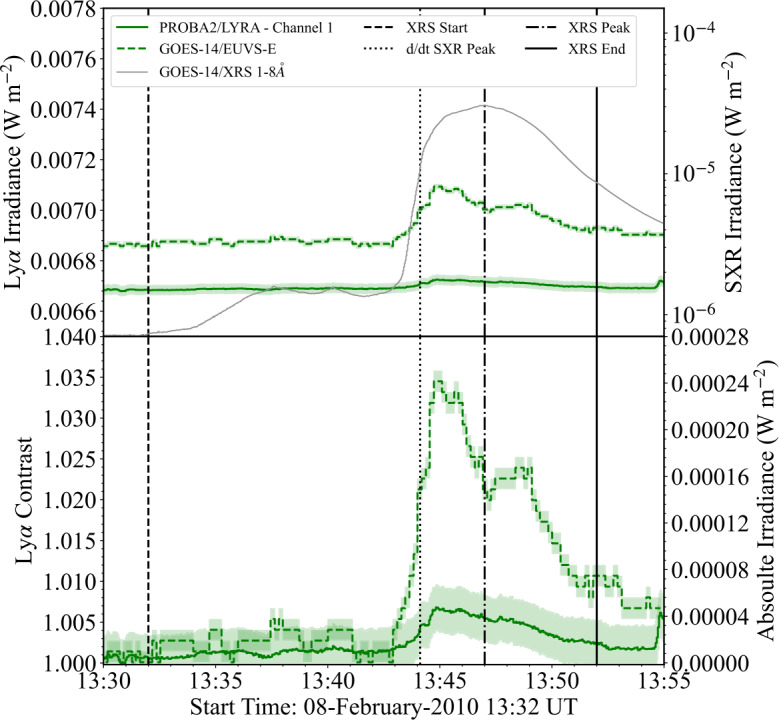
Flare lightcurves from the analysis of SOL2010–02–08. (Top) Ly$\alpha $ and SXR irradiance lightcurves from GOES-14/EUVS–E (green dashed), GOES-14/XRS (grey solid), and PROBA2/LYRA (green solid). (Bottom) Normalised Ly$\alpha $ enhancement and excess flux of Ly$\alpha $ emission for each instrument. In both panels, the green dotted line denotes the PROBA2/LYRA data following manual calibration of L1 data. The shaded regions denote the data $\pm \sigma $, where $\mathrm{\sigma }$ is the standard deviation of the flux in a 600 s preflare window for each observation. Vertical dashed, dotted–dashed, and solid lines denote the XRS start, XRS peak, and XRS end times, respectively. Vertical dotted lines denote the peak of the SXR derivative.

The M2.0 flare that occurred on 8 February 2010 was jointly observed by GOES-14/EUVS-E and PROBA2/LYRA. An image from the *Sun Watcher using Active Pixel System detector and Image Processing* (SWAP: Berghmans et al. 2006) onboard PROBA2 is presented in Figure 5 and the raw lightcurves from both instruments are presented in top panel of Figure 6, while the contrasts and excess fluxes are shown in the bottom panel. From Figure 6 it is apparent that there is a substantial disagreement between the relative fluxes from GOES-14/EUVS–E and PROBA2/LYRA, with the flare profile from PROBA2/LYRA appearing notably lower in comparison to GOES-14/EUVS–E. The peak value of the relative flux was found to be $\mathrm{7.1 }\cdot \mathrm{10^{-3}~Wm^{-2}}$ and $\mathrm{6.7}\cdot \mathrm{10^{-3}Wm^{-2}}$ for GOES/EUVS–E and PROBA2/LYRA, respectively. The respective peaks in Ly$\alpha $ from the two instruments appear to show temporal agreement with each other but not the SXR derivative. Kretzschmar, Dominique, and Dammasch (2013) conducted a detailed analysis of this flare and found similar results in the flux, suggesting this is due to delayed brightening in the EUV wavelengths as the flare plasma cools and is therefore coronal in origin.

From the bottom panel of Figure 6, the peak contrasts in Ly$\alpha $ were found to be approximately 3.5% and 0.7% for GOES-14/EUVS–E and PROBA2/LYRA, respectively, demonstrating a factor of five difference in their calculated values. Moreover, the peak value of excess flux was found to vary from $\mathrm{2.4 }\cdot \mathrm{10^{-4}~Wm^{-2}}$ and $\mathrm{0.5 }\cdot \mathrm{10^{-4}~Wm^{-2}}$ between observations. Converting the excess flux to units of power and integrating over the full flare period, the total energy radiated in Ly$\alpha $ as observed by GOES-14/EUVS–E was found to be $\mathrm{1.3 }\cdot \mathrm{10^{29}~erg}$, three times larger than that found for PROBA2/LYRA, which was calculated as $\mathrm{0.4 }\cdot \mathrm{10^{29}~erg}$. Such a difference in total energy becomes significant to calculations of the chromospheric energy budget when compared with HXR spectroscopic observations.

One explanation for the significant difference in contrast and flare excess between GOES-14/EUVS-E and PROBA2/LYRA could be contamination of the LYRA bandpass from continuum emission during QS conditions. Specifically the response function of unit 2 of PROBA2/LYRA (used for this study) contains an additional feature at approximately 2000 Å, which is beyond the range of Figure 2. The “out-of-band” continuum emission in this range accounts for approximately 70% of the observed QS emission in this unit. Despite this, during flare conditions the enhancements in emission in this spectral range are minimal. Thus, the relative flare signal measured by PROBA2/LYRA in this case can be considered to be dominated by Ly$\alpha $. However, when discussing contrast and flare excess it is likely that the continuum emission in the preflare signal may contribute to a large background flux, which in turn leads to a reduced contrast and flare excess in the overall measurements from PROBA2/LYRA (M. Dominique 2024 – private communication).

### SOL2016–04–18

**Figure 7:**
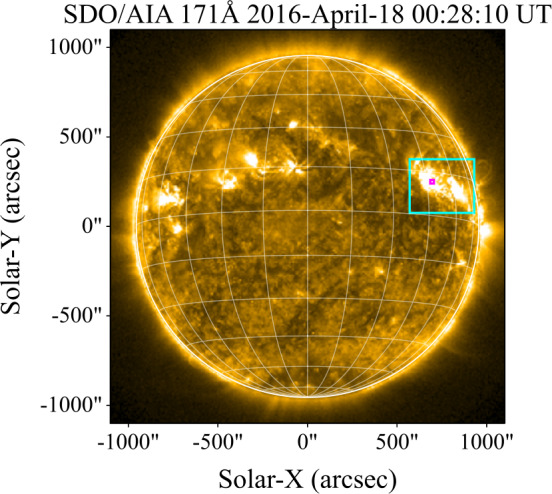
SDO/AIA (171 Å) image of SOL2016–04–18 at a time close to the flare peak. The cyan bounding box denotes the active region location. The small magenta box denotes the approximate position of the flare source.

**Figure 8:**
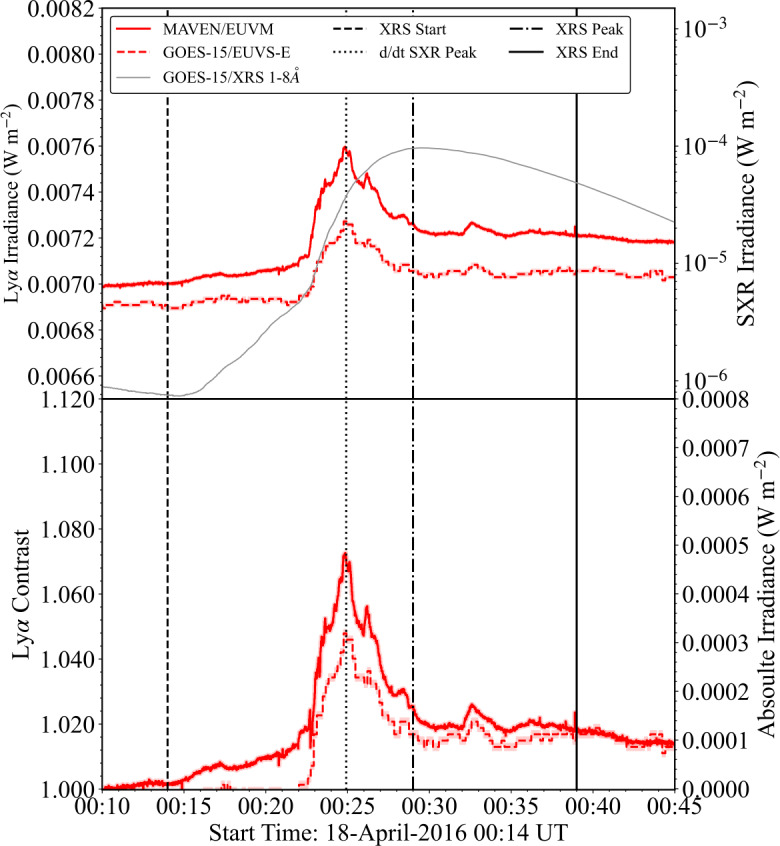
Flare lightcurves from the analysis of SOL2016–04–18. (Top) Ly$\alpha $ and SXR irradiance lightcurves from GOES-15/EUVS–E (red dashed), GOES-15/XRS (grey solid), and MAVEN/EUVM (red solid). (Bottom) Normalised Ly$\alpha $ enhancement and excess flux of Ly$\alpha $ emission for each instrument. The shaded regions denote the data$\pm \mathrm{1\sigma }$, where $\mathrm{\sigma }$ is the standard deviation of the flux in a 600 s preflare window for each observation. Vertical dashed, dotted-dashed, and solid lines denote the XRS start, XRS peak, and XRS end times, respectively. Vertical dotted lines denote the peak of the SXR derivative.

The M6.7 flare on 18 April 2016 was co-observed in Ly$\alpha $ from the Earth and Mars by GOES-15/EUVS–E and MAVEN/EUVM, respectively. Imaging for this flare from SDO/AIA (171 Å) is presented in Figure 7. Lightcurves from both observations (following the standardisation of the MAVEN/EUVM data and CLV correction) are presented in the top panel Figure 8. The peak relative flux was found to be within 4% between GOES-15/EUVS–E and MAVEN/EUVM. The observations show remarkable temporal agreement. Slight differences in their temporal behaviour only occur around short bursts, which are captured by MAVEN/EUVM but not GOES-15/EUVS–E due to the factor of 10 difference in their cadences. The contrasts and excess fluxes calculated from each observation are presented in the bottom panel of Figure 8. The peak contrasts for GOES-15/EUVS–E and MAVEN/EUVM were found to be 4.2% and 7.4%, respectively. The peak values of the excess flux were found to be within a factor of 1.5, with corresponding total energies of $\mathrm{3.0 }\cdot \mathrm{10^{29}}$ and $\mathrm{5.5 }\cdot \mathrm{10^{29}~erg}$. Thus, the energy found using MAVEN/EUVM is almost a factor of two larger than that of GOES-15/EUVS–E.

### SOL2023–05–09

**Figure 9:**
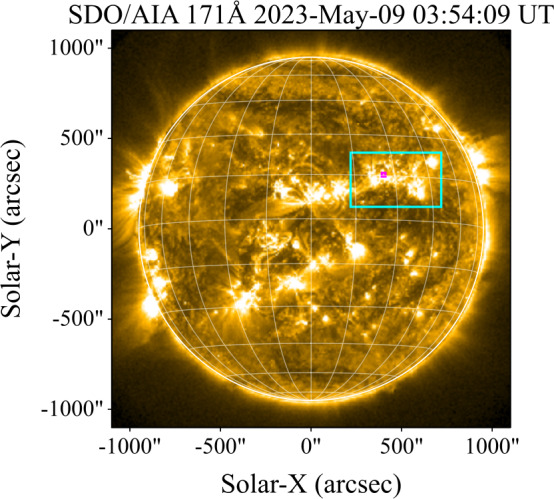
SDO/AIA (171 Å) image of SOL2023–05–09 at a time close to the flare peak. The cyan bounding box denotes the approximate flare location. The small magenta box denotes the approximate position of the flare source.

**Figure 10:**
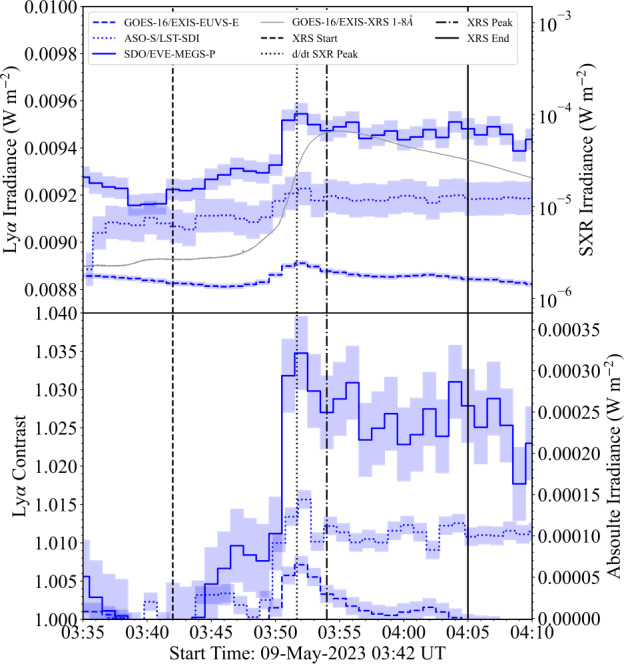
Flare lightcurves from the analysis of SOL2023–05–09. (Top) Ly$\alpha $ and SXR irradiance lightcurves from GOES-16/EXIS–EUVS–B (blue dashed), GOES-16/EXIS–XRS (grey solid), ASO-S/LST-SDI (blue dotted), and SDO/EVE-MEGS-P (blue solid). (Bottom) Normalised Ly$\alpha $ enhancement and excess flux of Ly$\alpha $ emission for each instrument. The shaded regions denote the data$\pm \mathrm{\sigma }$, where $\mathrm{\sigma }$ is the standard deviation of the flux in a 600 s preflare window for each observation. Vertical dashed, dotted–dashed, and solid lines denote the XRS start, XRS peak, and XRS end times, respectively. Vertical dotted lines denote the peak of the SXR derivative.

The M6.5 flare on 9 May 2023 was observed photometrically in Ly$\alpha $ by both GOES-16/EXIS–EUVS–B and SDO/EVE-MEGS-P, as well as imaged by ASO-S/LST-SDI. A context image from SDO/AIA (171 Å) is presented in Figure 9. Following the radiometric calibration of the ASO-S/LST-SDI data detailed in Section 4.1.2, comparison was made with the photometric observations. The top panel of Figure 10 presents the relative flux from each instrument. It is apparent that peak relative fluxes for the three observing instruments differ by approximately 6%. The larger flux found SDO/EVE-MEGS-P may be attributed to broadening of the instrument bandpass over time, which is not necessarily measurable (Woodraska 2023 – private communication).

Despite the similarity in flare profiles, the contrasts were found to range from 0.7% to 3.5%. This suggests that despite the similarity in the measured Ly$\alpha $ peak, the sensitivity to the QS flux levels may drive discrepancies in the background values with which the contrasts are calculated. Similarly, the peak excess fluxes differed by up to $\mathrm{2.5}\cdot \mathrm{10^{-4}~Wm^{-2}}$ between all observations, translating to a maximum discrepancy in total energy of $\mathrm{4.0 }\cdot \mathrm{10^{29}~erg}$.

### Timings

**Figure 11:**
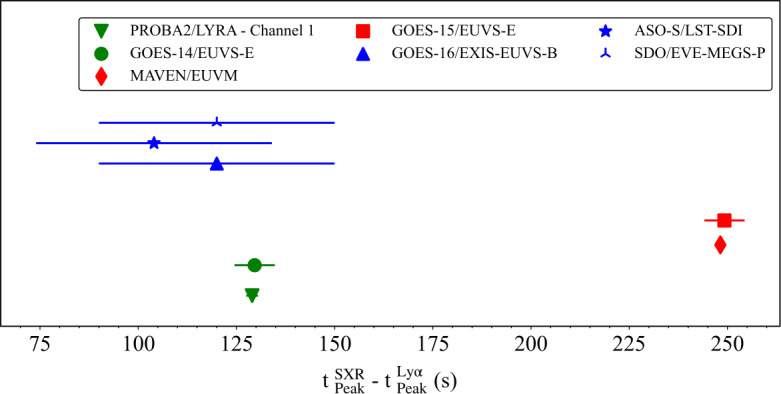
Time differences between the SXR peak (1 – 8 Å) from GOES and the Ly$\alpha $ peak for each observing instrument for each flare. Colours denote each flare in the sample. Markers denote the unique observing instrument. The width of the horizontal lines affixed to each marker correspond to the instrument cadence.

For each flare, the time difference between the GOES SXR peak in the 1 – 8 Å band and the observed Ly$\alpha $ peak from each instrument was calculated in order to identify any lag or lead times. Figure 11 presents the absolute value of $\mathrm{t^{SXR}_{Peak} }-\mathrm{ t^{Ly\alpha}_{Peak}}$ ($\Delta \mathrm{t_{Peak}}$) for each instrument. The uncertainty is taken as $\pm \mathrm{\frac{cadence}{2}}$, given by the horizontal error bars. For SOL2010–02–08 there is a disparity of 0.6 s between the $\Delta \mathrm{t_{Peak}}$ values calculated for PROBA2/LYRA and GOES-14/EUVS–E. There is a remarkable agreement between the $\Delta \mathrm{t_{Peak}}$ values calculated for GOES-15/EUVS–E and MAVEN/EUVM for SOL2016–04–18 with a discrepancy of 0.3 s. For SOL2023–05–09, there is a broad agreement in $\Delta \mathrm{t_{Peak}}$ between GOES-16/EXIS–EUVS–B and SDO/EVE-MEGS-P, although the uncertainty of these values is particularly large due to the relatively low (60 s) cadence between measurements. There is a time difference of 16 s in $\Delta \mathrm{t_{Peak}}$ between these two instruments and ASO-S/LST-SDI. However, the $\Delta \mathrm{t_{Peak}}$ values for this flare all lie within the associated uncertainties of one another. Generally, the peak times between instruments are in relatively good agreement with each other given the associated uncertainties in the recorded times.

## Discussion and Future Missions

In this work, we present an inter-instrument comparison of flare-related Ly$\alpha $ observations for three M-class flares, focussing on the relative and excess flux, the calculated flare contrast, and the total radiated energy. From this we find measurement inconsistencies to exist to a varying degree across seven different Ly$\alpha $ instruments. The key findings are as follows:

- Relative fluxes between instruments for all three flares examined here are in sufficient agreement, such that the conclusions drawn from their analysis will be negligibly impacted by the choice of instrument.
- Discrepancies in the contrasts and excess fluxes are of sufficient degree to have a substantial effect on the conclusions drawn from multi-instrument Ly$\alpha $ studies.
- The calculated energies between instruments are sufficiently different to potentially have a significant impact on constraining the contribution of Ly$\alpha $ to the radiated energy budget of solar flares.
- The flare timings between instruments are in relatively good agreement considering the uncertainties and therefore are minimally impacted by the choice of observing instrument.

Milligan et al. (2020) demonstrated that 95% of M- and X-class flares observed by GOES-15/EUVS–E have an associated Ly$\alpha $ contrast of $\leq 10\%$, with an upper limit to the contrast of $\sim 30\%$, while Raulin et al. (2013) found Ly$\alpha $ contrasts measured by PROBA2/LYRA to be consistently $<1\%$. The flares examined here were found to have contrasts ranging from 0.3% up to 7.4%, but importantly it has been demonstrated here that these ranges may be influenced by the observing instrument. The findings presented here show that the contrast range may vary by up to $\sim 3.5\%$ depending on the observing instrument. This discrepancy is over half of the range of contrast variability found in Greatorex, Milligan, and Chamberlin (2023). Moreover, it has been shown that the calculated values of excess flux can vary by up to a factor of five between instruments. This implies that analyses of solar flare excess in Ly$\alpha $ may be significantly impacted by the choice of observing instrument, thus influencing the conclusions drawn from flare observations and further calculations of the flare energy carried out using these fluxes. On the contrary, the values of relative flux were found to be in sufficient agreement such that any analysis done with these fluxes would be minimally impacted by the discrepancies between observations from different instruments.

Calculations of the radiated energy in Ly$\alpha $ are also vital for estimating the contribution of this wavelength (and therefore others) to the radiated energy budget of the chromosphere. Relatively few studies have compared the radiated energy in Ly$\alpha $ to the incident nonthermal electron energy deposited into the chromosphere during flares. From those studies, Ly$\alpha $ has been found to radiate up to $\sim 8\%$ of the chromospheric energy (Milligan et al. 2014). Here it has been demonstrated that calculations of the radiated energy in Ly$\alpha $ may vary by up to an order of magnitude between observations from different instruments. This has significant implication for statistical studies attempting to quantify the radiated energy in Ly$\alpha $ over a large flare sample, particularly when using multiple instruments as part of those observations. In this work, it has not been possible to compute the nonthermal electron energies for each flare due to a lack of uniform HXR observations from a single observing instrument. Introducing variation in HXR observations will add unnecessary excess uncertainty in calculated energies that may distract from the discrepancies in the Ly$\alpha $ observations. Under the assumption of a single nonthermal energy for each flare, the discrepancies in the calculated Ly$\alpha $ energy could substantially alter estimates of the percentage contribution of Ly$\alpha $ emission to the radiated energy budget of the chromosphere.

Data driven models such as FISM2 depend on observations to form statistics, from which approximations can be made for emission spectra from solar flares. These models have application in the study of atmospheric responses to solar flares (Qian et al. 2010, 2011, 2012; Lollo et al. 2012), as well as in the development of models designed to aid in the analysis of observations from missions such as MAVEN (Chaffin et al. 2015; Chaufray et al. 2015; Jain et al. 2015; Sakai et al. 2016; Thiemann et al. 2017). The interpretation of the results from studies employing these models is thus reliant on the precision and credibility of the underlying observations.

Significant disagreement in the observed Ly$\alpha $ peak times between GOES/EUVS–E and SDO/EVE-MEGS-P were presented by Milligan and Chamberlin (2016). They found a delay in peak times between instruments of 5 – 10 minutes, which was eventually attributed to the Kalman filter used to smooth data in SDO/EVE processing; this filter was subsequently replaced with a Fourier transform filter, which ultimately prevented the recurrence of this journal. The time discrepancies presented in this work are not as substantial as those presented in Milligan and Chamberlin (2016); the differences in peak times between instruments could be attributed to the variability in cadences, or in the case of imagers, the assumptions of constant flux between exposure, which may be an oversimplification of the true behaviour of the Ly$\alpha $ flux. While temporal discrepancies between observations may not have a substantial impact on calculations flare energetics, they may affect the understanding of energy transport processes in flares as well as the derived relationships between solar flares and associated atmospheric responses in the unique sub-regions of the ionosphere (Raulin et al. 2013; Berdermann et al. 2018; Milligan et al. 2020; Hayes et al. 2021; Chakraborty et al. 2021; Barta et al. 2022).

The convolution of the FISM2 spectra in Section 3 demonstrates that there is minimal contribution to the total measured Ly$\alpha $ irradiance from additional species in the bandpass of each instrument. Minor variations between instruments are present in the wings of the Ly$\alpha $ profile as well as in the blueward and redward wavelengths surrounding the Ly$\alpha $ line. However, the core emission significantly dominates the measured flux in all cases by at least two orders of magnitude, therefore the total measured irradiance may be considered to be predominantly from the Ly$\alpha $ line.

Given the standardisation of the observations carried out in this work, it is implicit that the observed discrepancies found between instruments are unlikely to be attributable to the observation field of view or observing techniques of the instruments. Instead, the discrepancies in the measured flux between instruments may be driven by inherent properties of the instrument itself or the calibration process carried out on the raw data. Absorption from the goecorona may be able to account for differences in observed flux between instruments at different orbital heights. Specifically, satellites in the Low-Earth Orbit may measure lower levels of Ly$\alpha $ irradiance compared to those with more extended orbits due to the absorption of Ly$\alpha $ photons by Hydrogen in the Earth’s geocorona. Wauters et al. (2022) suggested that geocornal absorption may be a potential explanation for a factor of 20 difference between GOES-15/EUVS-E and PROBA2/LYRA observations of a solar flare in Ly$\alpha $. Additionally, the spectral assumptions used to scale observations for each instrument may influence the returned flare profiles. For example, GOES-15/EUVS-E data are scaled to the *Whole Heliosphere Interval* QS reference spectrum and thus systematic uncertainties may be present in the observed flare data (Woods et al. 2009; Milligan 2021).

Ultimately, it is important to acknowledge any discrepancy in observations between instruments, particularly when conducting multi-instrument studies, which are becoming evermore possible with the expanding availability of flare-related Ly$\alpha $ observations. Previously, GOES observations in the SXRs have been considered the “industry standard” for flare classifications and timings. It may be of value to establish some form of agreeable standard of observation to scale measurements from dedicated Ly$\alpha $ missions to. Analysis of results from upcoming missions with Ly$\alpha $ observing capabilities such as Solar-C featuring the Solar Spectral Irradiance Monitor (SoSpIM: Harra et al. 2022), and the *The Solar eruptioN Integral Field Spectrograph* (SNIFS: Chamberlin et al. 2020b) sounding rocket will benefit from recognition of the diversity of conclusions that may be drawn from Ly$\alpha $ observations depending on the observing instrument. The findings presented here may guide interpretation of the observations taken by the current and new generation of Ly$\alpha $ instruments as part of future studies.

## Data Availability

No datasets were generated or analysed during the current study.

## Footnotes

1 SO was omitted from this study due to difficulty in obtaining sufficient calibration data.

2 Due to an instrumentation failure on 26 May 2014, MEGS-A is no longer operational.

3 This has increased to 16.5 s due to degradation of the instrument.
